# A theoretical model for optimal control of banana Moko ( *Musa* AAB Simmonds)

**DOI:** 10.12688/f1000research.27373.2

**Published:** 2021-02-19

**Authors:** Marly Grajales Amorocho, Anibal Muñoz Loaiza

**Affiliations:** 1 Grupo de Investigaciones en Biodiversidad y Biotecnologia, Universidad del Quindío, Armenia, Quindío, Colombia; 2 Grupo de Modelación Matemática en Epidemiología, Facultad de Educación, Universidad del Quindío, Armenia, Quindío, Colombia

**Keywords:** Mathematical model, Moko, Banana, Ralstonia solanacearum, Logistic growth

## Abstract

A population simulation model with non-linear ordinary differential equations is presented, which interprets the dynamics of the banana Moko, with prevention of the disease and population of susceptible and infected plants over time. A crop with a variable population of plants and a logistic growth of replanting is assumed, taking into account the maximum capacity of plants in the delimited study area. Also, with the help of farmers, the costs of implementing prevention strategies and elimination of infected plants were calculated per week in order to determine the optimal conditions that control the disease and reduce production costs. We found that the implementation of prevention strategies *(f)* plays an important role, but the parameter that most influences the threshold value is the elimination of infected plants *g* *. * However, to reduce production costs due to the high implementation of prevention strategies and to maintain the disease in a controlled state, both controls *u* ^*1*^ **and *u* ^*2*^ should be implemented between 40% and 60%, obtaining with this percentage an approximate reduction of 51.37% in production costs per week, where in 23 weeks following the same conditions it is expected to have a healthy plantation without infected plants.

## Introduction

The banana is a fruit of great economic importance and food sovereignty, because it is found in the shopping basket of people across different social strata and because of its nutritional content. However, its production is threatened by re-emerging diseases such as Moko, caused by the bacterium *Ralstonia solanacearum* race 2 philotype II ( Fegan & Prior, 2006), which causes wilting and deterioration of the plant. Symptoms are usually visible when there is a great spread of bacteria and adjacent plants may have already been infected ( Viljoen *et al.*, 2016). Moko is a peculiar manifestation of bacterial wilt; it is a quarantine pest that, once inside the host, moves through the vascular bundles. Being a vascular disease, this bacterium not only affects the vegetative part but also the daughter hill, promoting the spread of the disease, which is accelerated by the high optimal minimum, optimal and maximum temperatures of 10°C, 35°C and 41°C, respectively, infecting triploid plantains, heliconia ( *Heliconia* spp.) and other ornamental Musacea plants ( Jiang *et al.*, 2017).

The development of mathematical models has contributed through the use of a wide range of techniques to the study of epidemics and diseases, helping to answer biological questions and raising new questions related to the epidemiology and ecology of pathogens and the diseases they cause; in most cases, mathematical models lead directly to applications in the control of the disease ( Jeger *et al.*, 2018). In plants, some prediction models have evaluated the control of some diseases in plantain and bananas, such as banana wilt by *Xanthomonas* (BXW) using mathematical models, describing a deterministic SI-type epidemic model for control of BXW focusing on the integrated management of the disease through cultural control as in Nannyonga *et al.* (2015), who considered the optimal control strategies associated with the prevention of transmission by the use of contaminated tools. The researchers assumed a model with three modes of transmission: vertical (from the mother plant to its child), horizontal (indirect) from the vector to plant, and through contaminated agricultural tools (Nannyonga *et al.*, 2015).

Likewise, Nakakawa *et al.* (2016) presented a mathematical model for BXW propagated by an insect vector. The mathematical model they formulated takes into account inflorescence infection and vertical transmission from the mother corm to the daughter hills, but not tool-based transmission by humans ( Nakakawa *et al.*, 2016). In this context, a dynamic system is formulated based on ordinary two-dimensional differential equations that interprets the dynamics of incidence of banana Moko disease, including prevention and treatment.

In the research carried out by ( Bautista-Montealegre *et al.*, 2016), the state of the disease in 2016 is shown, these authors to contribute to the management of banana Moko disease in the department of Quindío-Colombia, evaluated the relationship between the incidence of the disease and variables related to physical and chemical properties of the soil, as well as the use of the soil and the altitudinal location in 269 farms analyzing soils and foliar tissues, as well as the symptoms of the disease to establish the effect of the variables on the probability of occurrence of the disease, finding a positive and significant correlation between the incidence of the disease, the hydraulic conductivity and the saturation of potassium in the soil; and negative and significant with the altitude, foliar copper concentration and presence of associated crops; Likewise, they argue that 10 of the 12 municipalities in the department have a high percentage of the disease, demonstrating the inadequate phytosanitary management that is still being carried out ( Bautista-Montealegre *et al.*, 2016).

## The model

Mathematical models are a tool of a growing scientific branch and of a notorious and marked interdisciplinary nature, linking mainly biologists and mathematicians, but also researchers from other areas with the challenge of applying mathematical techniques to the study of biological processes ( García-Macías & Ubertini, 2019). Previously, they were used mainly in epidemiology in SI, SIS, SIR, SIRS epidemic models among others; at present, mathematical models are, therefore, a useful tool in biology, agronomy, phytopathology, chemistry, environment and among many other areas, since they allow to make a representation of a biological system, the behavior of a certain disease etc., and with them facilitate the understanding of its dynamics in order to make predictions for future decisions on actions that facilitate control ( Jeger *et al.*, 2018).

The model presents the following assumptions:

- In the plantation the total plant population is assumed to be positive.
- It is considered a plantation with a maximum capacity of banana plants *k*
- It is considered that the disease of the banana Moko, follows a model Epidemic type SI (Susceptible-Infectious).
- Banana plants in asymptomatic state are not considered.
- Removal of infected plants is considered.

**Figure 1.**
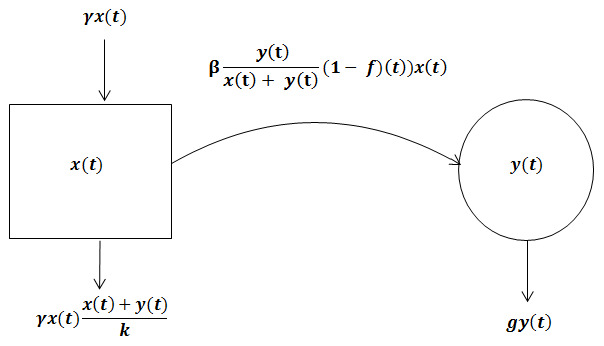
Banana Moko's disease diagram with prevention.

A population model with nonlinear ordinary differential equations is presented, which interprets the dynamics of the banana Moko, including a constant rate of disease prevention in the population of susceptible plants over time. A variable population of plants and a logistic growth of replanting are assumed, taking into account the maximum capacity of plants in the study region. The variables and parameters of the model are: *x*( *t*), the average number of susceptible banana plants; *y*( *t*), the average number of diseased banana plants; and *P*( *t*) = *x*( *t*) + *y*( *t*), total number of banana plants at one time *t*, shown in Figure 1.

The model parameters are: *γ*, constant overseeding rate; *k*, load capacity (maximum capacity) of banana plants in the study region; and β, probability of transmission of infection. Preventive controls are: *g*, fraction of infected banana plants removed; and *f*, fraction of susceptible banana plants that receive prevention of contagion of the bacteria. The dynamic system that interprets the infectious process including prevention and elimination, is formed by the following two nonlinear differential equations:

$$\frac{dx(\text{t})}{dt}=\gamma \left(1-\frac{x(\text{t})+y(\text{t})}{k}\right)x(\text{t})-\beta \frac{y(\text{t})}{x(\text{t})+y(\text{t})}(1-f)x(\text{t})\equiv h(.)\;(1)$$

$$\frac{dy(\text{t})}{dt}=\beta \frac{y(\text{t})}{x(\text{t})+y(\text{t})}(1-f)x(\text{t})\;-gy(\text{t})\equiv \omega \;(.)\;(2)$$

With initial conditions *x*(0) = *x*_0_, *y*(0) = *y*_0_, *P*(0) = *x*(0) + *y*(0), *γ*, *k* > 0, 0 < *f*, *g*, β < 1, *P* ≤ *k*, *x*(t) ≡ *x*, and *y*(t) ≡ *y*.

The region of eco-epidemiological sense is defined where the trajectories of the plant infection dynamics make sense,

$$\Omega =\{(x,\;y)\;\in \;R_{+}^{2}\;:\;x\;+\;y\;\leq \;k\}.$$

In the first equation, the derivative $\frac{dx}{dt}$ indicates the variation of the susceptible banana plant population with respect to time in weeks *t*, which is given by the inflow, the growth of susceptible banana plants by replanting γ *x* regulated by the factor $1-\frac{x+y}{k}$ minus the outflow, the incidence of banana plants *λ*( *y*)(1 – *f*) *x*, where $\lambda (y)=\text{β}\frac{y}{x+y}$ is the infection force of banana Moko and (1 – *f*) is the fraction of population of susceptible banana plants on which no preventive measures were taken.

 Similarly, in the second equation the derivative $\frac{dy}{dt}$ represents the variation of the population of infected plants with respect to time in weeks *t*, given by the inflow the incidence of banana plants minus the outflow the population of plants infected deleted *gy*.

## Stability analysis

We start by finding the equilibrium populations, the constant solutions of the system, where the population variation of susceptible plants and variation of infected plants become zero, that is, $\frac{dx}{dt}=0;\frac{dy}{dt}=0$

$$\left\{\gamma \left(1-\frac{x+y}{k}\right)-\frac{\beta (1-f)y}{x+y}\right\}x=0\;(3)$$

$$\left\{\frac{\beta (1-f)x}{x+y}-g\right\}y=0\;(4)$$

We solve this non-linear algebraic system for *x* and *y*, determining the equilibrium point according to agricultural conditions.

From Equation (4) *y* = 0 o

β(1−f)x=g(x+y)(5)

Substituting *y* = 0 in Equation (3), we obtain

$$\gamma \left(1-\frac{x+y}{k}\right)\;x=0$$

Of which *x* = 0 or *x* = *k*. Therefore, we have the point *E*_0_ = (0,0), which does not make agricultural sense, since *P* > 0 and the equilibrium point *E*_1_ = ( *k*,0), disease free and in which the susceptible population equals the capacity maximum.

Solving for *y* from Equation (5), we obtain

$$y=\frac{[\beta (1-f)-g]x}{g}\;(6)$$

Substituting Equation (6) in Equation (3), we obtain the components of equilibrium point with disease, *E*_3_ = $(\hat{x},\hat{y}).$

$$\hat{x}=\frac{k}{\frac{\beta (1-f)}{g}}\left(1-\frac{\text{β}\;\left(\frac{\beta (1-f)}{g}-1\right)(1-f)}{\text{γ}\frac{\beta (1-f)}{g}}\right),\;\hat{y}=\frac{k\;\left(\frac{\beta (1-f)}{g}-1\right)}{\frac{\beta (1-f)}{g}}\left(1-\frac{\text{β}\;\left(\frac{\beta (1-f)}{g}-1\right)(1-f)}{\text{γ}\frac{\beta (1-f)}{g}}\right)$$

Considering,

$$\xi_{0}=\frac{\beta (1-f)}{g}\;\text{and}\;\rho \;\text{=}\;\frac{\text{β}\;(\frac{\beta (1-f)}{g}-1)(1-f)}{\text{γ}\frac{\beta (1-f)}{g}}=\frac{\text{β}\;(\xi_{0}-1)(1-f)}{\text{γ}\xi_{0}},$$

We write *x* and *y* like this

$$\hat{x}=\;\frac{k}{\xi_{0}}\;(1-\rho ),\;\hat{y}=\frac{\;k(\xi_{0}-1)}{\xi_{0}}\;(1-\rho )$$

In coexistence of populations $\hat{x}>0$ and $\hat{y}>0$, which is true when ξ _0_ > 1 and ρ < 1.

Since *P* = *x* + *y*, the total plant population in equilibrium is, $\hat{P}=\hat{x}+\hat{y}.$ That is,

$$\hat{P}=\;\frac{k}{\xi_{0}}\;(1-\rho )\;+\frac{\;k(\xi_{0}-1)}{\xi_{0}}\;(1-\rho )$$

Therefore, $\hat{P}=k\;(\rho -1).$

*ξ*_0_, indicates the average number of infected plants that an infected plant produces during the infectious period (before being killed) in the population of susceptible plants and is considered the threshold of infected plants. We can consider this threshold as a function that depends on *f* and *g*,

$$\xi_{0}(f,\;g)=\frac{\beta (1-f)}{g}.$$

To determine the stability of each equilibrium point (E), we apply the Hartman-Grobman theorem ( Perko, 2008), linearizing the system of non-linear Equation (1) – Equation (2), obtaining the linearization matrix (Jacobian matrix) of the form:

$$J(E)=\left(\begin{array}{cc} h_{x}(\text{E}) & h_{y}(\text{E})\\ \omega_{x}(\text{E}) & \omega_{y}(\text{E}) \end{array}\right)\;(7)$$

With the following partial derivative elements,

$$a_{11}=h_{x}(E)=\gamma -\frac{\gamma }{k}(\hat{x}\;+\;\hat{y})-\frac{\gamma }{k}\hat{x}-\frac{\beta (1-f){\hat{y}}^{2}}{{\left(\hat{x}+\hat{y}\right)}^{2}}$$

$$a_{12}=h_{y}(E)=-\frac{\gamma }{k}\hat{x}\;-\frac{\beta (1-f){\hat{x}}^{2}}{{\left(\hat{x}+\hat{y}\right)}^{2}}$$

$$a_{21}=\omega_{x}(E)=\frac{\beta (1-f){\hat{y}}^{2}}{{\left(\hat{x}+\hat{y}\right)}^{2}}$$

$$a_{22}=\omega_{y}(E)=\frac{\beta (1-f){\hat{x}}^{2}}{{\left(\hat{x}+\hat{y}\right)}^{2}}-g$$

These elements of the matrix J (E) are the coefficients of the linear system

$$\frac{d}{dt}\text{U}=J(E)U$$

Where, *U* = ( *u, v*) ^*t*^ (transposed vector).

We analyze the **equilibrium** points with an agricultural sense *E*_1_ = ( *k*, 0) y *E*_3_ = $(\hat{x},\hat{y}).$ For *E*_1_, we obtain the Jacobian matrix,

$$J(E_{1})=\left(\begin{array}{cc} \lambda & -\left\{\gamma +\beta (1-f)\right\}\\ 0 & g(\xi_{0}-1) \end{array}\right)$$

Because it is a triangular matrix, the eigenvalues ( *λ _i_*, *i* = 1,2)

$$\lambda_{1}=-\gamma ,\;\lambda_{2}=g(\xi_{0}-1)\;$$

where *λ*_2_ < 0 since the threshold *ξ*_0_ < 1. 

We conclude that the free equilibrium point of Moko disease is locally and asymptomatically stable.

For case $E_{3}=(\hat{x},\;\hat{y}),$ in matrix ( 7) we obtain the trace and the determinant of *J*( *E*_3_), respectively,

$$Traz.J(E_{3})=a_{11}+a_{22}\;;\;det.J(E_{3})=a_{11}a_{22}-a_{12}a_{21}$$

We conclude that the equilibrium point with susceptible plants and infected plants is locally and asymptomatically stable if the threshold inequalities ( *ξ*_0_ > 1 *and* *ρ* < 1) and the inequalities are met,

$$\gamma +\frac{\beta (1-f){\hat{x}}^{2}}{{\left(\hat{x}+\hat{y}\right)}^{2}}<\frac{\gamma }{k}(\hat{x}+\hat{y})+\frac{\gamma }{k}\hat{x}+\frac{\beta (1-f){\hat{y}}^{2}}{{\left(\hat{x}+\hat{y}\right)}^{2}}+g$$

$$\left\{\frac{\gamma }{k}\hat{x}+\frac{\beta (1-f){\hat{x}}^{2}}{{\left(\hat{x}+\hat{y}\right)}^{2}}\right\}\frac{\beta (1-f){\hat{y}}^{2}}{{\left(\hat{x}+\hat{y}\right)}^{2}}>\left\{\gamma \;-\;\frac{\gamma }{k}\;(\hat{x}+\hat{y})\;-\;\frac{\gamma }{k}\;\hat{x}\;-\;\frac{\beta (1-f){\hat{y}}^{2}}{{\left(\hat{x}+\hat{y}\right)}^{2}}\right\}\left[g\;-\;\frac{\beta (1-f){\hat{x}}^{2}}{{\left(\hat{x}+\hat{y}\right)}^{2}}\right]$$

**Figure 2.**
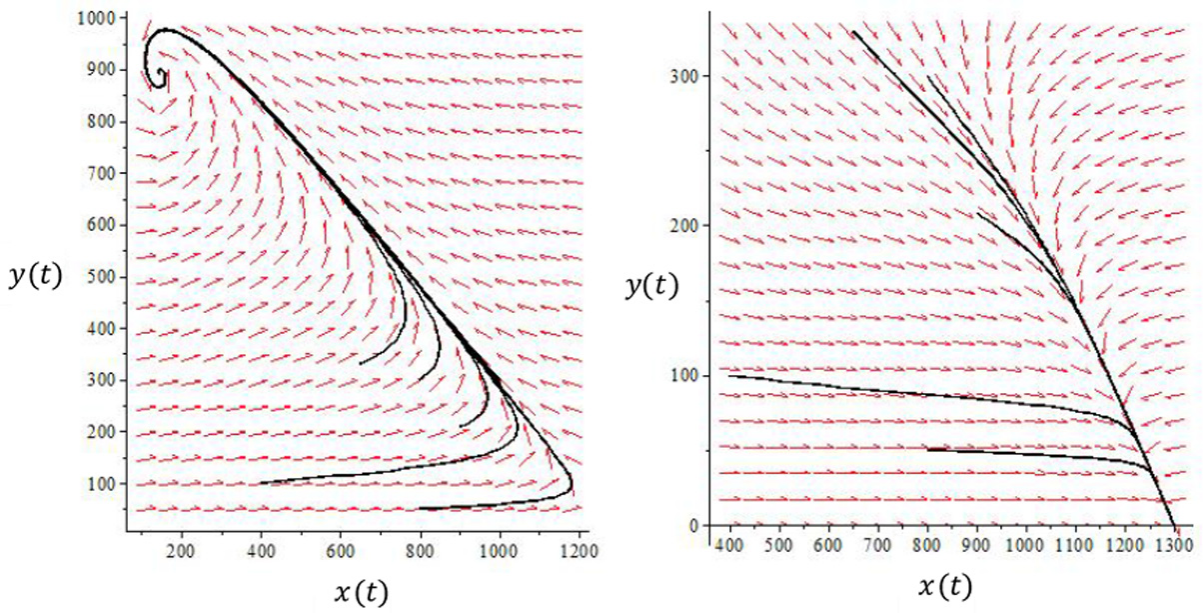
Local stability of the susceptible plant population and infected plant population corresponding to *ξ*_0_ = 7 y ξ _0_ = 0.79.

These analytical results are shown in the phase planes of Figure 2, made with Maple 18 software ( free trial available; SageMath is an openly available alternative), for different scenarios varying initial conditions.

## Sensitivity analysis

The local sensitivity analysis was performed that is a measure of the relative change in a variable when its parameters change ( Chitnis *et al.*, 2008; Hamby, 1994; Rodrigues *et al.*, 2013). That is,

$$I_{\xi_{0}}^{p}=\frac{\partial \xi_{0}}{\partial p}\frac{p}{\xi_{0}}$$

Where,

$$\;\xi_{0}=\frac{\beta (1-f)}{g}$$

y *p*: *β*, *g*, *f*, are previously defined parameters.

The indices of local sensitivity of the threshold ${\text{ξ}}_{0}^{p}$ were calculated with respect to each parameter:

$${\text{I}}_{\xi_{0}}^{\beta }=\frac{\left(1-f\right)}{g}\;\frac{\beta }{\frac{\beta (1-f)}{g}}=1$$

$$I_{\xi_{0}}^{f}=\frac{-\beta }{g}\;\frac{f}{\frac{\beta (1-f)}{g}}=\frac{-f}{1-f}$$

$${\text{I}}_{\xi_{0}}^{g}=\frac{\beta (1-f)}{g^{2}}\;\frac{g}{\frac{\beta (1-f)}{g}}=-1$$

**Figure 3.**
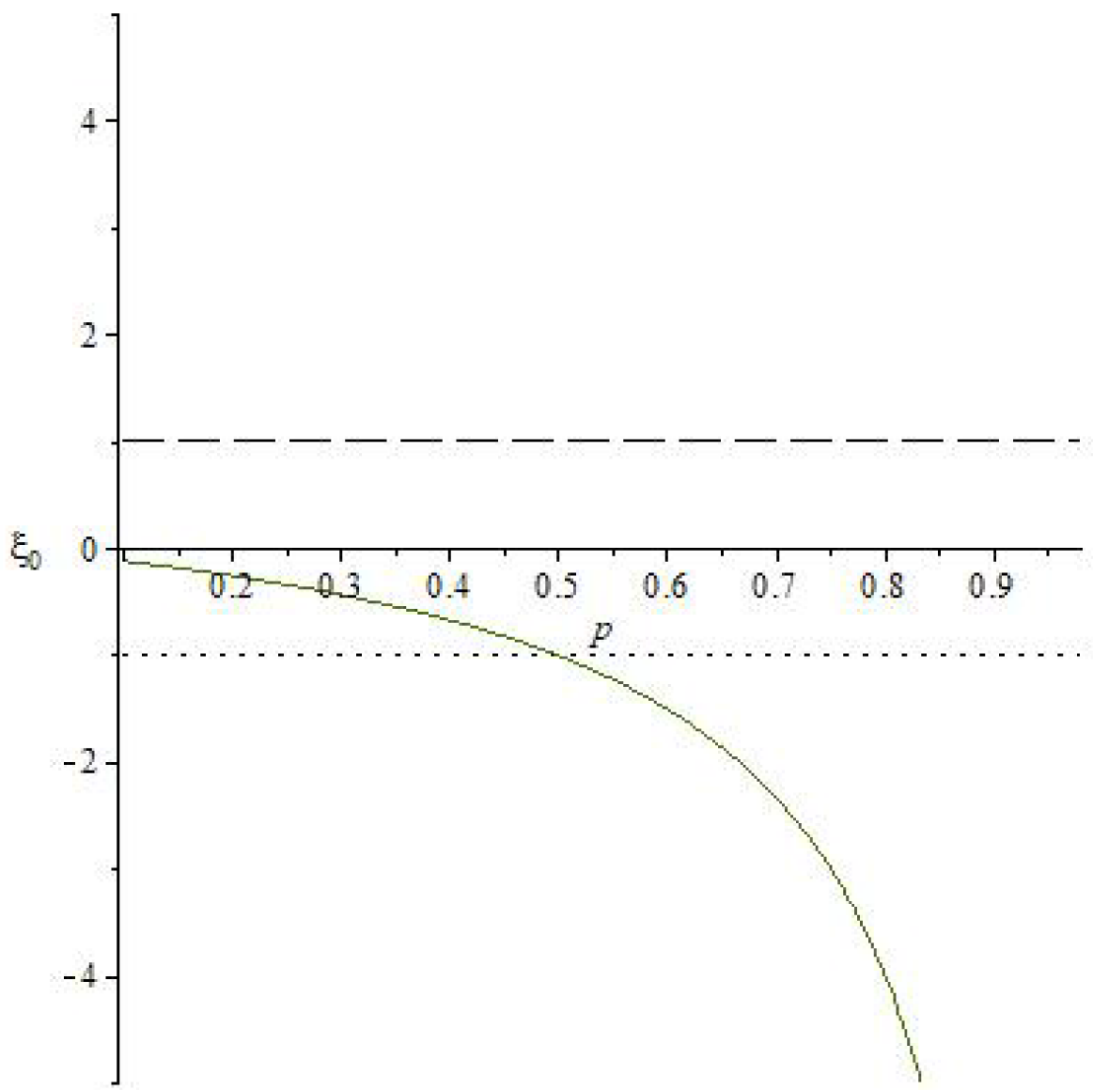
In the graph the line (∙∙∙∙∙∙) corresponds to the index $I_{\xi_{0}}^{g}$, the line (---) at the index $I_{\xi_{0}}^{\beta }$ and the line ( ______ ) at the index $I_{\xi_{0}}^{f}$, *p* indicates each parameter *g,β and f*.

With respect to the values of the threshold indices of the disease and the Figure 3., the following observations are made:

- For *f* = 0.5 it is true that $I_{\xi_{0}}^{g}$ = $I_{\xi_{0}}^{f}$
- When the percentage of plants receiving prevention increases, the disease threshold decreases, that is, they are inversely proportional. This behavior is good in managing the disease.
- For values of *f,g* < 0.5, the disease threshold index is lower for the elimination of infected plants ( *g*) and in the case that *f,g* > 0.5, the disease threshold index is lower in the case of prevention of susceptible plants.
- The disease threshold increases proportionally with respect to the transmission probability.

It is concluded that mathematical simulation models are a useful tool for research in banana Moko disease. With them it was determined that the elimination of banana plants infected with the disease plays an essential role in the good agronomic management of the crop.

## Optimal control problem

**Figure 4.**
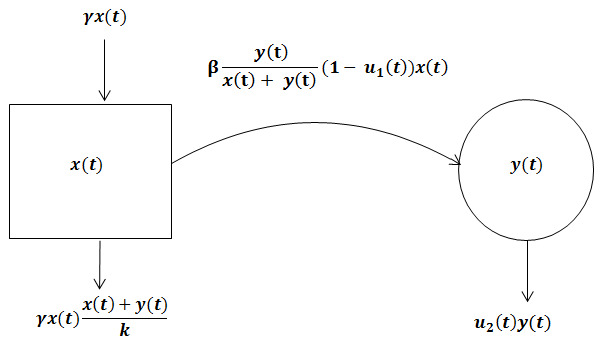
Dynamics of banana Moko with variable controls.

An objective, quadratic and cost functional linked to a system is presented of nonlinear ordinary differential equations, which interprets the dynamics of banana Moko ( Figure 4), including a constant rate of disease prevention in the population of susceptible plants over time. It assumes a variable population of plants and a logistic growth of replanting having taken into account the maximum capacity of plants in the study region ( Cherruault & Gallego, 1985; Louadj *et al.*, 2018). The variables and parameters of the optimal control problem are described in Table 1.

The functional objective of direct and indirect costs is proposed:

$$J(x,u)=\int_{0}^{T}L(x,u)dt=\int_{0}^{T}\left\{\eta_{1}y(t)+\frac{\eta_{2}}{2}u_{1}^{2}(t)\;+\frac{\eta_{3}}{2}u_{2}^{2}(t)\right\}\;dt$$

**Table 1.** Variables, parameters, parameter values, and initial populations at *t* = 0.

Variables, parameters	Description	Value	Reference
*x*( *t*)	Susceptible plants	800	assigned
*y*( *t*)	Infected plants	8	assigned
*p*( *t*) = *x*( *t*) + *x*( *t*)	Total plants	808	assigned
*k*	Loading capacity	1300	per hectare
β	Transmission probability	0.7	assigned
*γ*	Replanting rate	3	assigned
*u*_1_	Prevention practices	______	______
*u*_2_	Elimination of infected plants	______	______
*η*_1_	Costs for each *y*	10200	farmer
*η*_2_	Costs of applying *u*_1_	67260	farmer
*η*_3_	Costs of applying *u*_2_	51000	farmer

Linked to the system of differential equations:

$$\frac{dx(\text{t})}{dt}=\gamma \;\left(1-\frac{x(\text{t})+y(\text{t})}{k}\right)x(\text{t})-\beta \;\frac{y(\text{t})}{x(\text{t})+y(\text{t})}(1-u_{1}(t))x(\text{t})\equiv f_{1}$$

$$\frac{dy(\text{t})}{dt}=\beta \;\frac{y(\text{t})}{x(\text{t})+y(\text{t})}(1-u_{1}(t))x(\text{t})-u_{2}(t)y(\text{t})\equiv f_{2}$$

With initial conditions *x*(0) = *x*_0_, *y*(0) = *y*_0_, *P*(0) = *x*(0) + *y*(0), *γ*, *k* > 0, 0 ≤ *u*_1_, *u*_2_, β ≤ 1 and *P* ≤ *k*.

It is about finding optimal control $\left({\bar{u}}_{1}(t),\;{\bar{u}}_{2}(t)\right)$ such that:

$$J({\bar{u}}_{1}(t),{\bar{u}}_{2}(t))=\underset{\text{Γ}}{\min}J\;({\bar{u}}_{1}(t),{\bar{u}}_{2}(t))$$

Where,

$$\Gamma =\{({\bar{u}}_{1}(t),{\bar{u}}_{2}(t))\in L^{2}(0,\;T):0\leq \;u_{1}(t)\;\leq \;1,\;0\leq u_{2}(t)\;\leq \;1\}$$

is the space of admissible controls and *L* ^2^ is the space of integrable functions, and T is the control terminal time.

## Optimal control problem analysis

The Hamiltonian function or (Pontryagin function) is of the form:

$$H(x,u,\lambda )=L(x,u)+\sum_{i=1}^{2}\lambda_{i}f_{i}$$

where ***x*** = ( *x, y*) is the vector of state variables, ***u*** = ( *u*_1_; *u*_2_) the vector of controls, ***λ*** = ( *λ*_1_, *λ*_2_) the vector of attached or conjugate variables and ***L*** is the Lagrangian. That is to say,

$$H(x,u,\lambda )=\eta_{1}y(t)+\frac{\eta_{2}}{2}u_{1}^{2}(t)+\frac{\eta_{3}}{2}u_{2}^{2}(t)$$

$$+\lambda_{1}\left[\gamma \left(1-\frac{x(t)+y(t)}{k}\right)x(t)-\beta \frac{y(t)}{x(t)+y(t)}(1-u_{1}(t))x(t)\right]$$

$$\;+\lambda_{2}\left[\beta \frac{y(t)}{x(t)+y(t)}(1-u_{1}(t))x(t)-u_{2}(t)y(t)\right]$$

Applying the first order condition $\frac{\partial H}{\partial u_{1}}=0\;\text{y}\frac{\partial H}{\partial u_{2}}=0,$, the optimal control is obtained:

$$u_{1}(t)=min\left(max\left(0,\left(\frac{\lambda_{2}-\lambda_{1}}{\eta_{2}}\right)\frac{\beta \text{xy}}{x+y}\right),1\right)$$

$$u_{2}(t)=min\left(max\left(0,\left(\frac{\lambda_{2}y}{\eta_{3}}\right)\right),1\right)$$

The conjugate system (or adjoint system) has the form:

$$\frac{d\lambda }{dt}=-H_{x}(x,\lambda ,u)$$

that is to say,

$$\frac{d\lambda_{1}}{dt}=-\lambda_{1}A-\lambda_{2}C\equiv g_{1}(x,u,\lambda )$$

$$\frac{d\lambda_{2}}{dt}=-\eta_{1}-\lambda_{1}B-\lambda_{2}D\equiv g_{2}(x,u,\lambda )$$

Where,

$$A=\gamma -\frac{\gamma }{k}(x-y)-\frac{\gamma }{k}x-\frac{\beta (1-{\bar{u}}_{1})y^{2}}{{\left(x+y\right)}^{2}}$$

$$B\text{=}-\frac{\gamma }{k}x-\frac{\beta (1-{\bar{u}}_{1})x^{2}}{{\left(x+y\right)}^{2}}$$

$$C=\frac{\beta (1-{\bar{u}}_{1})y^{2}}{{\left(x+y\right)}^{2}}$$

$$D=\frac{\beta (1-{\bar{u}}_{1})x^{2}}{{\left(x+y\right)}^{2}}-{\bar{u}}_{2}$$

with transversality conditions *λ _i_*( *T*) = 0, *i* = 1,2.

The contour problem is formed by the system of state variables of the dynamics of the Moko with their respective initial conditions, the conjugated system and the terminal conditions and the optimal controls:

$$\{\begin{array}{c} \frac{dx}{dt}=F(x,\bar{u})\\ x(0)=x_{0}\\ \frac{d\lambda }{dt}=G(x,\bar{u},\lambda )\\ \lambda (T)=0\\ u_{1}(t)=min\left(max\left(0,\left(\frac{\lambda_{2}-\lambda_{1}}{\eta_{2}}\right)\frac{\text{βxy}}{x+y}\right),1\right)\\ u_{2}(t)=min\left(max\left(0,\left(\frac{\lambda_{2}y}{\eta_{3}}\right)\right),1\right) \end{array}$$

## Results and conclusions

**Figure 5.**
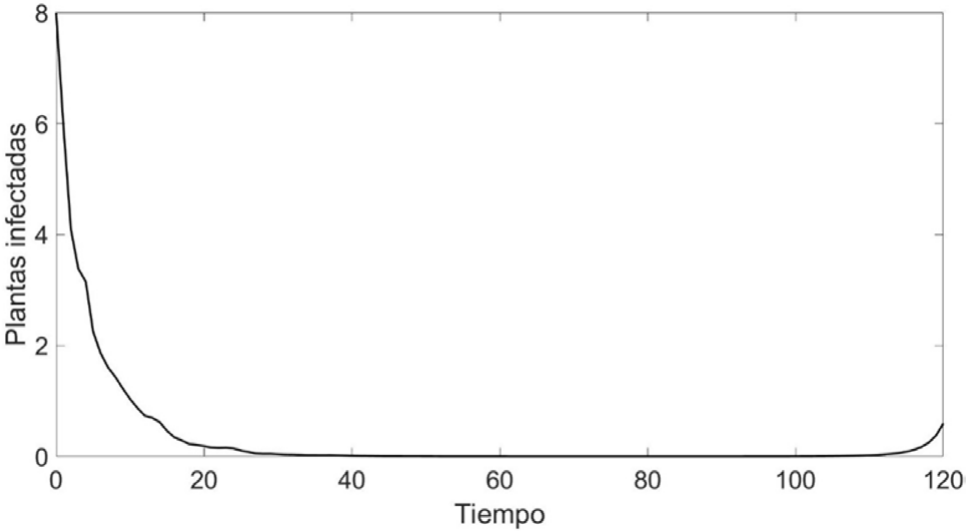
Behavior of infected plants of banana Moko disease in the time *t*.

**Figure 6.**
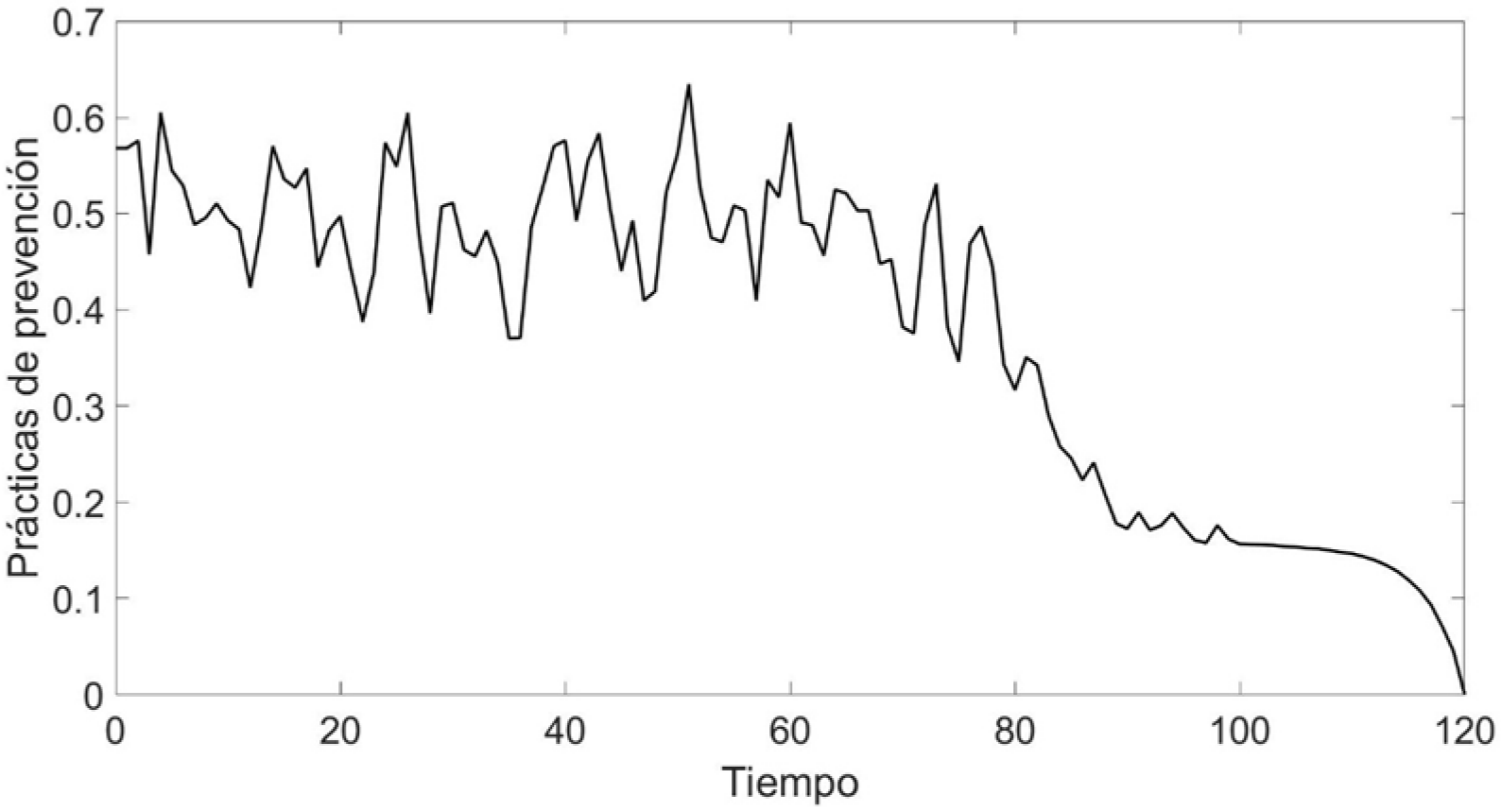
The trajectories of the control *u*_1_( *t*).

**Figure 7.**
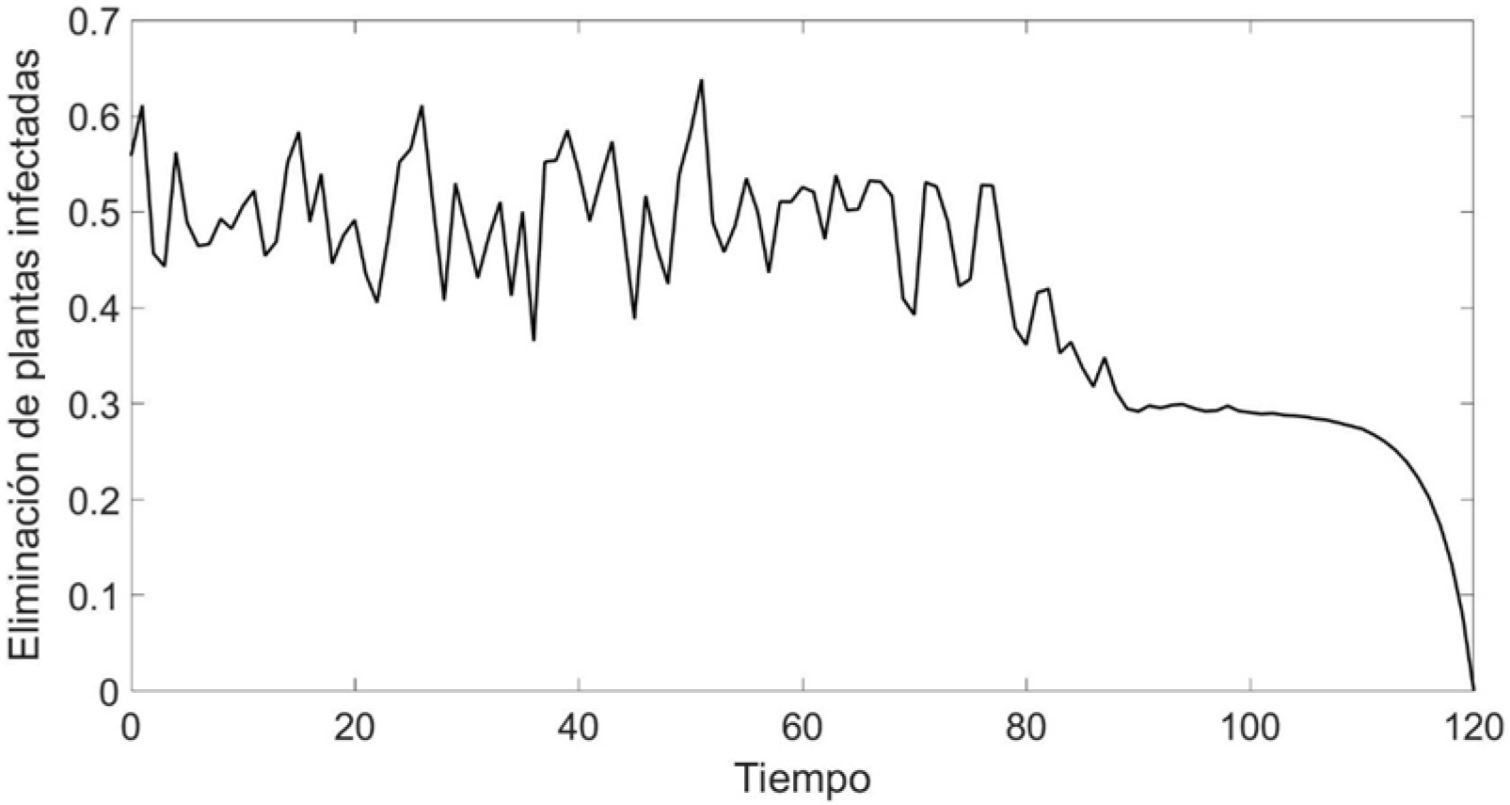
The trajectories of the controls *u*_2_( *t*).

Numerical analysis of the contour problem: with this analysis we can observe the decrease of the infected plants varying different conditions of the controls *u*_1_ and *u*_2_, showing that when *u*_1_ and *u*_2_ are not implemented as shown in the figure at week 120, the population of infected plants tends to increase ( Figure 5), and that the implementation of control *u*_1_ in approximately 45% ( Figure 6) and the implementation of control *u*_2_ in approximately 60% ( Figure 7), produces a complete decrease in infected plants at week 23.

With this we can determine that if both controls are implemented in 40% and 60% respectively, the banana Moko disease in a crop with good agronomic management tends to disappear, likewise it contributes to reducing the production costs associated with the loss of plants due to infection and the costs of implementing prevention strategies by 51.13% weekly, which is equivalent to 60,756 Colombian pesos.

We conclude that in order to reduce production costs and maintain the disease in a controlled state, the recommended prevention strategies should be implemented, and with greater relevance the detection and rapid elimination of infected plants.

## Data availability

All data underlying the results are available as part of the article and no additional source data are required.
