# Supplementary material for: A theoretical model for optimal control of banana Moko ( Musa AAB Simmonds)
Source: F1000Res. 2021 Feb 19;9:1443. Originally published 2020 Dec 10. [Version 2] doi: 10.12688/f1000research.27373.2 (PMC7921884; doi:10.12688/f1000research.27373.2)
Supplement: Peer review history

# Peer review: Reviewer response for version 2

**DOI:** 10.5256/f1000research.54612.r79924

**Published:** 2021-03-01

**Authors:** Dalia M. Muñoz

**Affiliations:** 1 Oceanographic Research Institute, Universidad Autónoma de Baja California, Ensenada, Mexico

This new version is clearer, more complete, and presents the relevance of the study.

 This version already includes more recent references.

 The optimal control section already appears as a complete and consistent section with the rest of the article.

 The labels of figures 5-7 should be written in English.

Is the work clearly and accurately presented and does it cite the current literature?

Partly

If applicable, is the statistical analysis and its interpretation appropriate?

Not applicable

Are all the source data underlying the results available to ensure full reproducibility?

Yes

Is the study design appropriate and is the work technically sound?

Yes

Are the conclusions drawn adequately supported by the results?

Partly

Are sufficient details of methods and analysis provided to allow replication by others?

Yes

Reviewer Expertise:

Public health, environment, sustainability, environmental management, econometrics

I confirm that I have read this submission and believe that I have an appropriate level of expertise to confirm that it is of an acceptable scientific standard.

# Peer review: Reviewer response for version 2

**DOI:** 10.5256/f1000research.54612.r79925

**Published:** 2021-02-24

**Authors:** Ana Maria Pulecio Montoya

**Affiliations:** 1 Department of Mathematics and Statistics, University of Nariño, Pasto, Colombia

The authors made all the suggested changes. In my opinion, the article is a great scientific contribution to understand the transmission dynamics of banana moko and also establishes control measures to guide farmers in the measures to be taken.

Is the work clearly and accurately presented and does it cite the current literature?

Partly

If applicable, is the statistical analysis and its interpretation appropriate?

Not applicable

Are all the source data underlying the results available to ensure full reproducibility?

Yes

Is the study design appropriate and is the work technically sound?

Yes

Are the conclusions drawn adequately supported by the results?

Partly

Are sufficient details of methods and analysis provided to allow replication by others?

Partly

Reviewer Expertise:

Mathematical biology, applied Mathematics

I confirm that I have read this submission and believe that I have an appropriate level of expertise to confirm that it is of an acceptable scientific standard.

# Peer review: Reviewer response for version 1

**DOI:** 10.5256/f1000research.30252.r76032

**Published:** 2021-01-04

**Authors:** Ana Maria Pulecio Montoya

**Affiliations:** 1 Department of Mathematics and Statistics, University of Nariño, Pasto, Colombia

The manuscript deals with analytical and numerical study of a model of Ordinary Differential Equations to treat banana Moko dynamics. I submit the following suggestions in order to improve this article:

- The abstract needs to explain the main results of the article.
- The authors should explain better the description in the differential equations of the model.
- The authors should explain better how they obtained the equilibrium points and why the equilibrium point E2 is not of interest in the analysis presented.
- Sensitivity analysis should be written as a section and the authors should explain better the results.
- The conclusions should highlight the main results of the model analysis without giving new results or repeating what has already been said.

Is the work clearly and accurately presented and does it cite the current literature?

Partly

If applicable, is the statistical analysis and its interpretation appropriate?

Not applicable

Are all the source data underlying the results available to ensure full reproducibility?

Yes

Is the study design appropriate and is the work technically sound?

Yes

Are the conclusions drawn adequately supported by the results?

Partly

Are sufficient details of methods and analysis provided to allow replication by others?

Partly

Reviewer Expertise:

Mathematical biology, applied Mathematics

I confirm that I have read this submission and believe that I have an appropriate level of expertise to confirm that it is of an acceptable scientific standard, however I have significant reservations, as outlined above.

# Peer review: Reviewer response for version 1

**DOI:** 10.5256/f1000research.30252.r76029

**Published:** 2020-12-23

**Authors:** Dalia M. Muñoz

**Affiliations:** 1 Oceanographic Research Institute, Universidad Autónoma de Baja California, Ensenada, Mexico

The authors present a simulation model to treat banana Moko dynamics, which constitutes an essential topic in food safety.  The model seems to be correctly implemented. However, the manuscript lacks some clarity, and more discussion on the results is strongly encouraged. The manuscript needs corrections before any decision. A point by point list with the concerns raised after my revision is listed in the next lines.

1. The abstract just mentioned how they performed the model and some other conditions like replanting. The abstract needs to be strengthened to discuss the main results and the implications of treating banana plants with some prevention measures.
2. The introduction needs to be strengthened to depict the state of the art on the banana Moko study. There exist other simulation models based on differential equations to treat the banana Moko? Why did you choose such a model?
  Addressing these aspects contributes to the authors pointing out the scarce literature in this regard and their study's relevance.
3. The following paragraph lacks clarity. Please rewrite it.
  Some prediction models that calculate the propagation threshold R0 have evaluated the control of some diseases in plantain and bananas, such as banana wilt by Xanthomonas (BXW) using mathematical models, describing a deterministic SI-type epidemic model for control of BXW focusing on the integrated management of the disease through cultural control as in Nannyonga et al. (2015), who considered the optimal control strategies associated with the prevention of transmission by the use of contaminated tools. The researchers assumed a model with three modes of transmission: vertical (from the mother plant to its child), horizontal (indirect) from the vector to plant, and through contaminated agricultural tools (Nannyonga et al., 2015).
4. The following sentences should be included in the section Model
  Local sensitivity is a measure of the relative change in a variable when its parameters change (Chitnis et al., 2008; Hamby, 1994; Rodrigues et al., 2013). That is….
5. What do authors mean when they talk about agronomic sense?
6. The results need to be expanded, including a better description. In the case of figure two, the authors do not present any description.
7. Conclusions generally present the main findings. Please rearrange it to meet the high-quality standards of the journal.
8. The presentation of the research perspective seems messy and needs to be improved. The authors present a research perspective, but it is not considered in the paper's main body. The author should consider it as part of the analysis. The optimal control problem should be in the Section Model, describe the development in the results and the implications or expected results with this analysis in conclusion.
9. The list of references requires an update to include recent studies.
10. Authors are encouraged to look for other models about diseases in banana crops and discuss their results to see advantages and differences.

Overall, the paper looks interesting, but it needs a major revision before any consideration.

Is the work clearly and accurately presented and does it cite the current literature?

Partly

If applicable, is the statistical analysis and its interpretation appropriate?

Not applicable

Are all the source data underlying the results available to ensure full reproducibility?

Yes

Is the study design appropriate and is the work technically sound?

Yes

Are the conclusions drawn adequately supported by the results?

Partly

Are sufficient details of methods and analysis provided to allow replication by others?

Yes

Reviewer Expertise:

Public health, environment, sustainability, environmental management, econometrics

I confirm that I have read this submission and believe that I have an appropriate level of expertise to confirm that it is of an acceptable scientific standard, however I have significant reservations, as outlined above.
